# A Low-Cost System Based on Image Analysis for Monitoring the Crystal Growth Process

**DOI:** 10.3390/s17061248

**Published:** 2017-05-31

**Authors:** Fabrício Venâncio, Francisca F. do Rosário, João Cajaiba

**Affiliations:** 1 Instituto de Química, Universidade Federal do Rio de Janeiro (UFRJ), Hélio de Almeida St., 40, Cidade Universitária, Rio de Janeiro 21941-614, Brazil; fabricioqv@ufrj.br; 2 Centro de Pesquisas e Desenvolvimento Leopoldo Américo Miguez de Mello, PETROBRAS, Cidade Universitária, Rio de Janeiro 21040-000, Brazil; frosario@petrobras.com.br

**Keywords:** imaging analysis, crystal growth, calcium carbonate

## Abstract

Many techniques are used to monitor one or more of the phenomena involved in the crystallization process. One of the challenges in crystal growth monitoring is finding techniques that allow direct interpretation of the data. The present study used a low-cost system, composed of a commercial webcam and a simple white LED (Light Emitting Diode) illuminator, to follow the calcium carbonate crystal growth process. The experiments were followed with focused beam reflectance measurement (FBRM), a common technique for obtaining information about the formation and growth of crystals. The images obtained in real time were treated with the red, blue, and green (RGB) system. The results showed a qualitative response of the system to crystal formation and growth processes, as there was an observed decrease in the signal as the growth process occurred. Control of the crystal growth was managed by increasing the viscosity of the test solution with the addition of monoethylene glycol (MEG) at 30% and 70% in a mass to mass relationship, providing different profiles of the RGB average curves. The decrease in the average RGB value became slower as the concentration of MEG was increased; this reflected a lag in the growth process that was proven by the FBRM.

## 1. Introduction

The observation of the onset of crystallization and monitoring of the crystal growth process are fundamental to understand crystal growth kinetics. Over the past two decades, several studies have concentrated on elucidating the mechanisms that govern crystallization processes. Reviews by Nagy et al. and Verma et al. [1,2] summarize the different techniques that have been used to monitor the onset of crystallization and crystal growth, shape, and size.

One of the most frequently used techniques for monitoring the crystal growth process is focused beam reflectance measurement (FBRM). This technique obtains information based on the number of crystals and produces a particle chord length distribution, which is a function of the true particle diameter distribution [3,4]. An important drawback of using an FBRM probe is that particles can accumulate in its window [1,5]. This phenomenon significantly alters the total particle count. 

Online imaging techniques can be used because they do not interfere in the crystallization process. Robust microscopy systems with algorithms for calculating crystal shape and size have been proposed for monitoring the crystal growth process [6,7,8,9,10,11]. Low-cost systems that have been proposed for watching crystallization phenomena are focused on detecting the onset of crystallization [12] or building solubility curves [13]. These online imaging techniques carry the advantage of having a large analysis area in the camera, thus mapping a suitable amount of the reaction media. The data provided are the primary colors red, blue, and green (RGB), as well as greyscale or average, the value of which varies from 0 to 255 [14].

In a qualitative approach, Sena et al. [15] used the RGB system to treat the images provided by a webcam to detect low concentrations of barium sulfate in a suspension and present the relationship between the RGB signal intensity and the solid concentration. Silva et al. [13] used the same imaging system to build solubility curves with a quantitative approach. These studies were focused on detecting the onset of crystallization.

This study used a low-cost online imaging system to follow the crystal growth process during calcium carbonate precipitation, while the viscosity of the test solution was manipulated so that we could observe changes in the crystal growth phenomenon. To verify the interpretation of the resulting signals, an FBRM probe was inserted in the reaction media to monitor the reaction.

## 2. Materials and Methods 

The experiments were performed on an Easymax 102 platform (Mettler Toledo, Columbus, OH, USA). A Microsoft Life Cam webcam was used to acquire the images and was configured to capture 24-bit digital images at a rate of 15 images/s and 1280 × 420 pixels of spatial resolution. From the images captured over time, the RGB absolute values’ components, provided by the RGBView software developed in the lab, were acquired. Illumination was provided by a white LED (Light Emitting Diode) with 730 lux of intensity, placed perpendicular to the camera. The supplementary material contains the parameters configured to the webcam used.

The results are provided in terms of RGB average, which was obtained through a simple average of the absolute values of the RGB components.

### 2.1. Influence of Different Particle Sizes on the Imaging System

Experiments were performed with three standard sizes of polymeric particles manufactured by the Coulter Corporation (Miami, FL, USA): 10 µm, 35 µm, and 500 µm. These experiments were conducted to measure the influence of particle size and rotation speed of the reaction media on the average values of the RGB components. The Easymax platform and the imaging system were used.

### 2.2. Calcium Carbonate Crystal Growth Experiments

Calcium chloride dehydrate, sodium bicarbonate, sodium chloride, and monoethylene glycol were used to prepare the test solutions. The concentrations of the solutions were 400 mg L^−1^ for calcium and 200 mg L^−1^ for bicarbonate. The salinity was increased by adding sodium chloride until the solution reached 40,000 mg L^−1^ of total chloride. The concentrations of monoethylene glycol used were 30% and 70% in a mass to mass relationship (*m*/*m*). All the solutions were filtered through a porous glass filter with a membrane mesh size of 0.45 µm.

**Figure 1:**
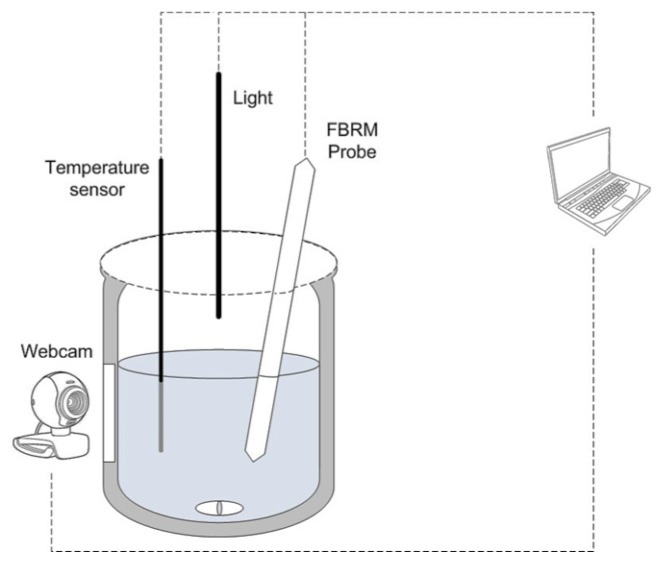
Schematic of the apparatus used to perform the experiments.

The 100-mL reactor vessel was stirred at a constant rate of 300 rpm, and the temperature was maintained at 80 °C during the experiments. The schematic in Figure 1 shows the apparatus used in the experiments.

The FBRM probe Model S400 (Mettler Toledo, Columbus, OH, USA) was inserted into the precipitation vessel to monitor particle size distributions in the range of 2 to 1000 µm, with a 2-s capture interval. To prevent the accumulation of the precipitate on the window of the FBRM probe, the probe was removed from the vessel every 8 min and cleaned. Data acquisition was started 2 min after each cleaning. For every 2 min of data acquisition, the averages of the reaction time and particle size distribution were recorded. These experiments lasted for 90 min.

## 3. Results and Discussion

The light was positioned perpendicular to the camera, as shown in Figure 1. When the light crossed the solution, part of it was reflected by the suspended particles and transmitted to the camera. Each small (micrometer-sized) particle showed the behavior of a punctual emission light source. Therefore, for particles of the same mass and type, the bigger the particles, the lower the amount of light reflected.

**Figure 2:**
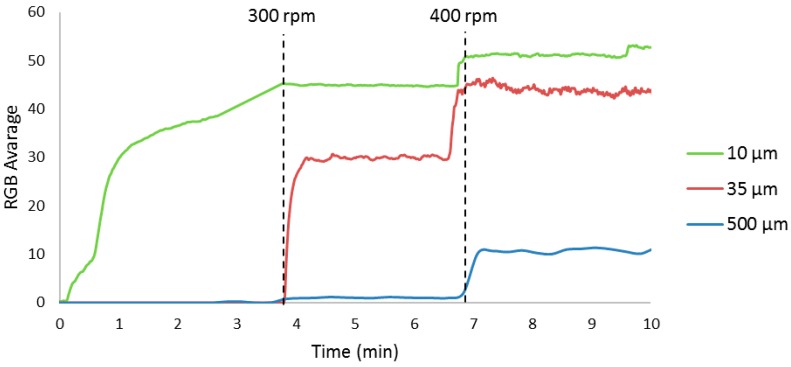
RGB averages of 0.1000 g of particles with three different sizes at 300 rpm (starts close to 4 min) and 400 rpm (starts close to 7 min).

Three experiments were performed to verify the response of the imaging system to three particle sizes and two stirring rates; the results are presented in Figure 2. As expected, the change in the rotation rate had an influence on the amount of light reflection.

As seen in Figure 2, smaller particles sizes produced higher average RGB values, and a higher rotation rate also increased the RGB values. Over the course of the crystallization process, the particle size changed due to crystal growth. Therefore, it is possible to use the RGB treatment system for monitoring the growth process of the crystals. 

**Figure 3:**
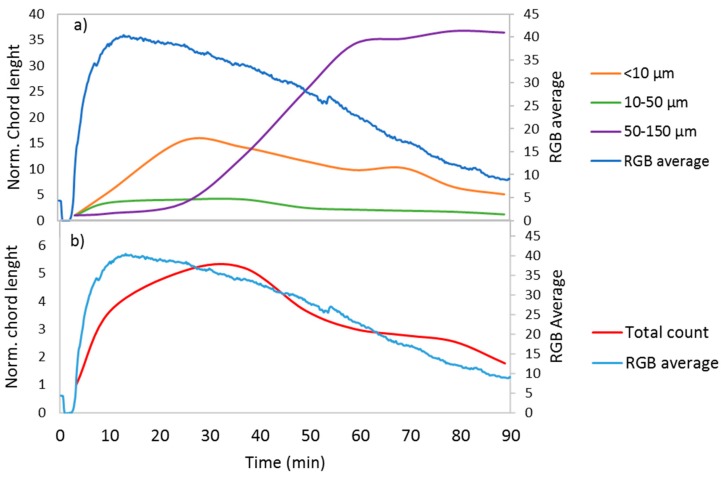
Normalized chord length and RGB average for a calcium carbonate precipitation process for (**a**) particles sized <10 µm, 10–50 µm, and 50–150 µm and (**b**) the total count.

Figure 3 shows the results of calcium carbonate precipitation monitoring by webcam and FBRM. The chord counts data were normalized in Figure 3 to better portray the modification of the components over time. The precipitation process began with an increase in the number of particles smaller than 50 µm (the groups of <10 µm and 10–50 µm in Figure 3a), followed by a decrease. The decrease of these particles was followed by an increase in the number of particles sized between 50 µm and 150 µm (Figure 3a), reflecting the growth of crystals. As can be seen in the figure, this process was followed by an increase of the RGB average at the beginning and a decrease of the average once the larger particles began to grow; the growth of crystals led to a decrease in the total particles (Figure 3b) and a decrease in the amount of reflected light.

**Figure 4:**
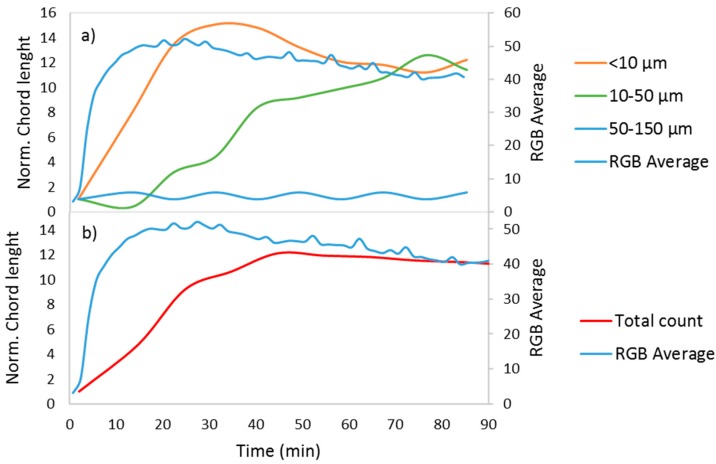
Normalized chord length and RGB average for a calcium carbonate precipitation process with 70% *m*/*m* of MEG for (**a**) particles sized <10 µm and 10–50 µm and (**b**) the total count.

To control the crystal growth process and prevent the formation of larger particles, the viscosity of the solution was increased by adding monoethylene glycol (MEG). Figure 4 shows the results of this process; particles sized between 50 µm and 150 µm were not formed.

**Figure 5:**
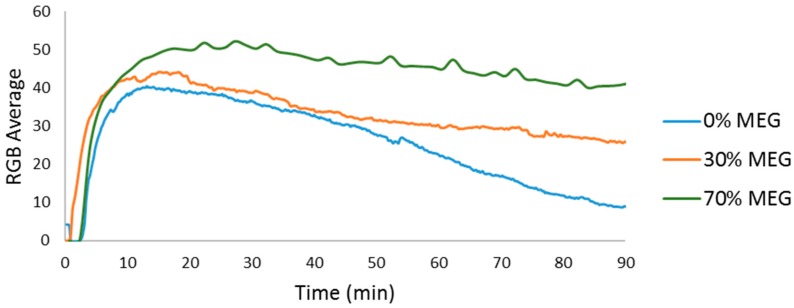
RGB average for calcium carbonate precipitation with three different concentrations of MEG.

As shown in Figure 4, the crystallization process began with an increase in the number of particles smaller than 10 µm. The decrease of these small particles was accompanied by an increase in the crystals sized between 10 µm and 50 µm, reflecting crystal growth (Figure 4a). This process was followed by an increase in the RGB average at the beginning and a decrease once the particles between 10 µm and 50 µm started increasing. However, the decrease of the RGB average was slower than the one shown in Figure 3; in Figure 4, the growth did not produce 50–150 µm particles, meaning more particles were present after the growth process and more light was reflected. In Figure 3, the difference between the RGB average at the peak and at the end of the experiment is 31, while Figure 4 shows a difference of 10. Figure 5 compares these RGB average signals and includes one more reaction with 30% *m*/*m* of MEG. As shown in the graph, the decrease is slower with an increasing amount of MEG, reflecting retarded growth and the presence of more particles.

The increase in MEG increased the supersaturation of calcium carbonate, producing more particles and higher RGB peak values than the experiments without MEG (Figure 5). It is possible to conclude—even without the reflectance measurements—that the system with 30% *m*/*m* MEG showed reduced crystal growth compared to the experiment without MEG and increased growth compared to the experiments that had 70% *m*/*m* MEG. 

The results show that it is possible to draw qualitative conclusions about crystal formation and the crystal growth process using our system. It is expected that with the optimization of the experiments, it will eventually be possible to perform quantitative analyses of different conditions and types of crystals with this system.

An increase in vaterite formation when the concentration of MEG was raised was observed. This result is in complete agreement with those presented by Flaten et al. [16]. The supplementary material provides the SEM images and XRD analysis of the experiments without and with 70% of MEG, as shown in Figure 3 and Figure 4. The RGB image analysis used cannot differentiate polymorphism probably because of the low resolution of the images obtained with the use of a simple webcam.

## 4. Conclusions

A qualitative analysis of calcium carbonate crystal growth was performed using a low-cost imaging system. Crystal growth was manipulated by increasing the viscosity of the test media, resulting in different curve profiles. With the optimization of our technique for different conditions and types of materials, a quantitative analysis of the crystal growth phenomenon is expected to be possible in the future. Furthermore, this system can also be applied to research on crystal growth models.

## Supplementary Materials

The Supplementary Materials are available online at http://www.mdpi.com/1424-8220/17/6/1248/s1.
