# Supplementary material for: A Low-Cost System Based on Image Analysis for Monitoring the Crystal Growth Process
Source: Sensors (Basel). 2017 May 31;17(6):1248. doi: 10.3390/s17061248 (PMC5491978; doi:10.3390/s17061248)
Supplement: Supplementary file 1 [file sensors-17-01248-s001.pdf]

sensors

# Supplementary Materials: A Low-Cost System Based on Image Analysis for Monitoring the Crystal Growth Process

**Fabrício Venâncio, Francisca F. do Rosário and João Cajaiba**

Webcam parameters: zoom 31x, brightness 144, white balance 5106, contrast 77, exposition −7, and saturation 7.

**Table S1.** SEM images.

<table>
<tr><th>MEG Content</th><th>2000x Amplification</th><th>6500x Amplification</th></tr>
<tr><td>Blank</td><td>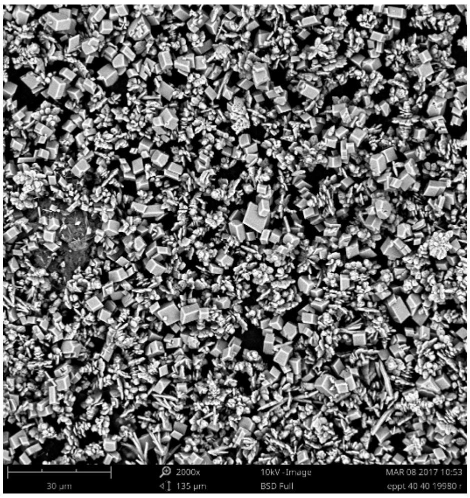
</td><td>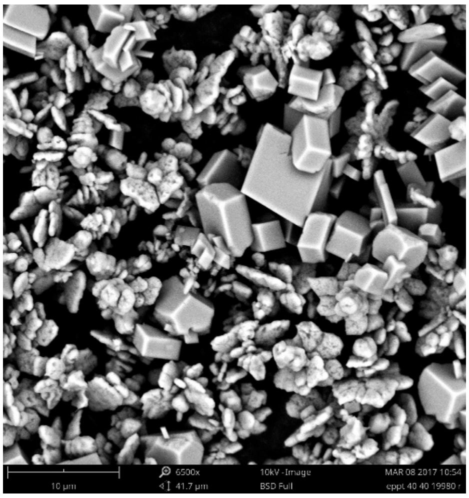
</td></tr>
<tr><td>60%</td><td>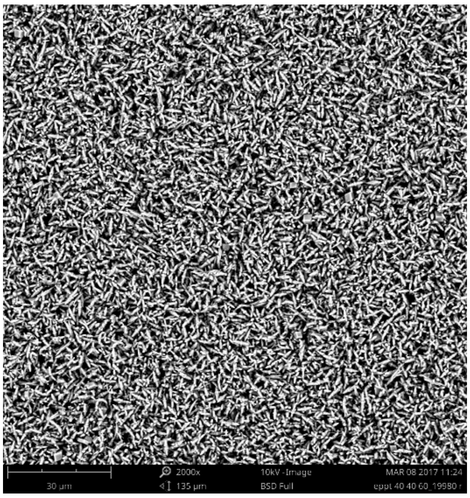
</td><td>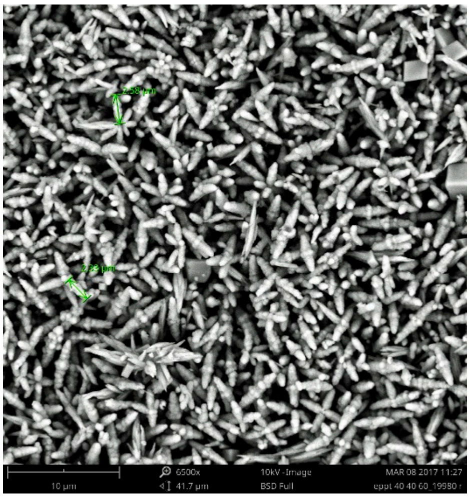
</td></tr>
</table>

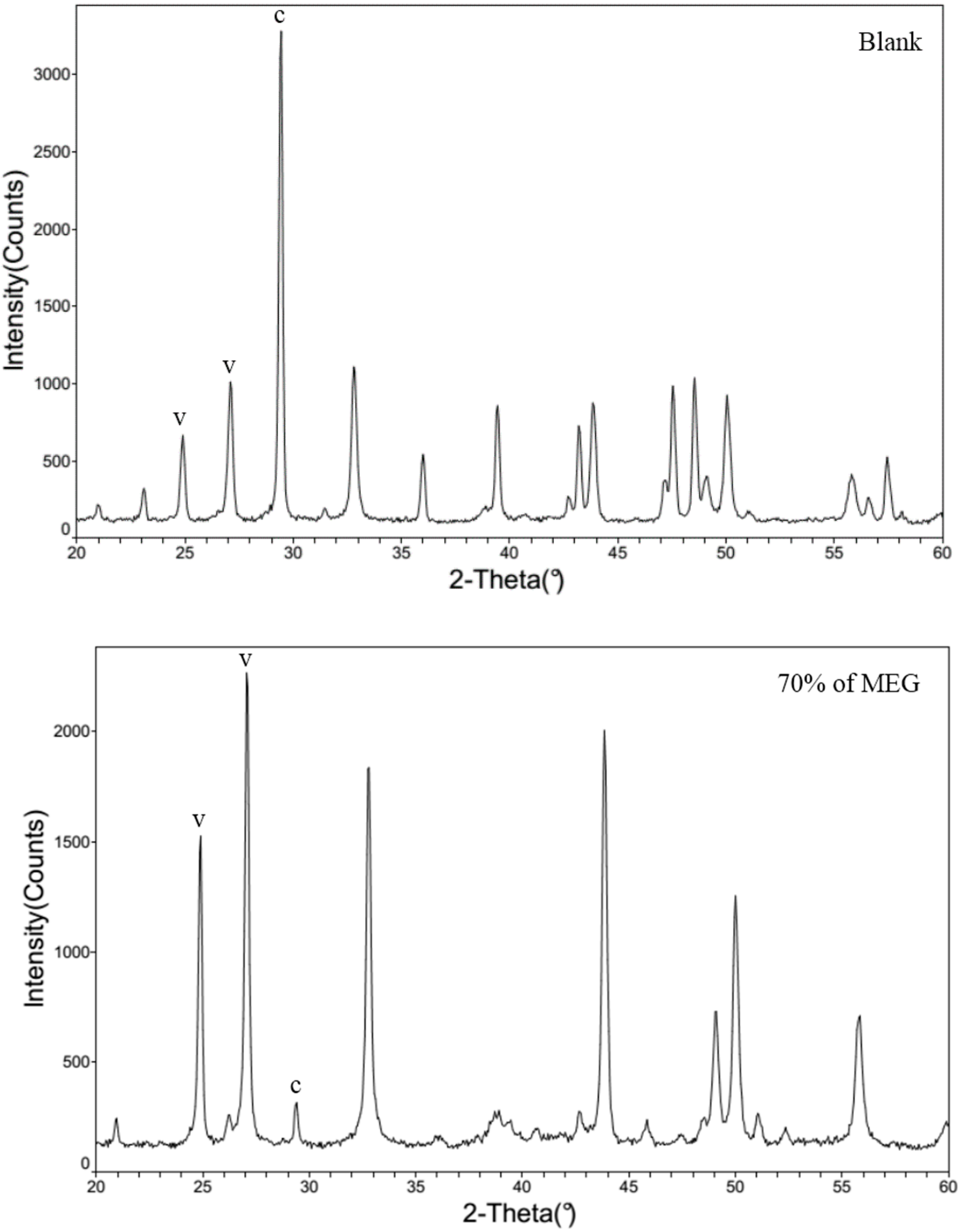


**Figure S1.** XRD analysis. Meaning: c- calcite; v- vaterite.
